# The landscape of disparities in obstetric neurocritical care and a path forward

**DOI:** 10.3389/fneur.2022.1008544

**Published:** 2023-01-06

**Authors:** Shilipi Mittal, Diana Alsbrook, Remi T. Okwechime, Farhana Iqbal, Christa O'Hana S. Nobleza

**Affiliations:** ^1^Department of Neurology, Vanderbilt University Medical Center, Nashville, TN, United States; ^2^Department of Neurology, University of Tennessee Health Science Center, Memphis, TN, United States; ^3^NeuroMedicine ICU, Critical Care Program University of Rochester Medical Center, Rochester, NY, United States; ^4^Maimonides Medical Center, Brooklyn, NY, United States; ^5^Baptist Memorial Hospital/Baptist Medical Group, Memphis, TN, United States

**Keywords:** obstetric care, neurocritical care, disparities health, pregnancy, health care

## Abstract

Health disparities in the obstetric population affect maternal morbidity and mortality. In the past years, there has been no significant improvement in disparities in care in the obstetric population. Patients who are pregnant are known to have a higher risk of pregnancy-associated neurologic conditions such as stroke and intracerebral hemorrhage. They can also experience concomitant neurocritical care disease states such as status epilepticus and traumatic brain injury. Studies exploring the disparities of care among pregnant patients who are neurotically ill are lacking. We aim to provide the landscape of disparities of care among the obstetric neurocritically-ill population and provide potential actionable opportunities to address these disparities in care.

## Introduction

A health disparity is defined as an increased burden of an adverse health outcome or health determinant within a specific subset of the population (1). These are directly related to inequality of race, ethnicity, economical and social status, and political and environmental aspects of the healthcare system. Racial and ethnic minority populations are projected to grow; thus, the impact of these disparities would become more evident and can affect outcomes (1). The cost of the race and ethnic health disparity in the United States (US) is about $245 M (2). Women face increased challenges in the setting of multi-level disparity in care due to societal and healthcare demands unique to them such as childbearing, child-rearing, and household responsibilities.

Several studies have demonstrated that severe maternal morbidity and maternal deaths are preventable by addressing racial and ethnic disparities (1). While it has also been shown that poverty, lack of education, poor nutritional status, smoking, alcohol abuse, and neighborhood have been associated with poor maternal and infant outcomes (3).

Pregnancy is known to increase the risk of cerebrovascular conditions such as ischemic stroke (IS), intracerebral hemorrhage (ICH), subarachnoid hemorrhage (SAH), and cerebral venous sinus thrombosis (CVST) (4). Patients who are pregnant can also be predisposed to seizures and status epilepticus (SE) (5). Due to the increased demands on the heart, pregnancy can also result in sudden cardiac arrest (6). Independent conditions can also cause traumatic brain injury (TBI) that can result in potential complications for the obstetric patient. The disparities in obstetric care and neurocritical care (NCC) and their effect on patient outcomes have not been widely explored. The aim of this review was to describe the current state of the disparities in obstetric and neurocritical care and to propose future directions to decrease the disparities in care.

### Disparities in obstetric and maternal care

Maternal mortality rates (MMRs) have been increasing over the last three decades in the US despite recent advances in healthcare technology and infrastructure. MMR is highest in the US among all the developed countries (7). From 2011 to 2015, the national pregnancy-related mortality rate (PRMR), defined as (while pregnant or within 1 year) was 17.2 per 100,000 live births. Black women and American Indian/Alaska Native women had the highest PRMRs (42.8 and 32.5, respectively), 3.3 and 2.5 times as high, respectively, compared to the PRMR for non-Hispanic White women (13.0). Hypertension in pregnancy, cardiomyopathy, and thromboembolic complications are significantly more common in Black women as compared to their White patient counterparts, and this disparity is worse in Black women aged 30–34 years. Hispanic women on other hand are more likely to die due to hemorrhage (8). Positive predictors of severe morbidity were also age < 20 and ≥30 years, self-pay or Medicaid coverage for delivery, low socioeconomic status, and presence of chronic medical conditions, but these factors do not completely explain the racial and ethnic disparities seen in maternal morbidity and mortality (8). Racial differences in the impact of inflammatory markers such as neutrophil-to-lymphocyte ratio (NLR), on the outcomes of neurologic, obstetric, and gynecologic patients, have been found as well (9–13). Among patients with stroke, it has been found that a higher NLR is seen among African Americans with both COVID-19 and stroke (9) while in women with adnexal torsion (AT), NLR is higher among Caucasians with AT vs. those with benign ovarian cysts without torsion, while for East Asian patients, there was no difference detected. Among patients with preeclampsia, a higher NLR ratio has been noted in severe cases (14). Another consideration in studying disparities in care is the genetic predisposition to malignancies that may occur concomitantly while pregnant. While there is increasing detection of brain metastasis among patients with breast cancer (15, 16), endocrine therapy responsiveness is becoming challenging, especially with resistance development reported up to 40% (15). The variability of therapy response according to race has been analyzed among hormone receptor-positive patients, and it has been shown that African American patients had a significantly higher risk of death than White patients (17). This disparity can be extrapolated toward obstetric patients with concomitant breast cancer and brain metastases and should be considered in treatment planning (18, 19). Overall, the multifactorial interplay of other factors such as educational background, access to clinical care (20), and pregnancy intent plays a role in disparity (21).

The divide in obstetric care also exists in patients in rural vs. urban populations, those with substance use disorder (SUD) and those who identify as LGBTQ (lesbian, gay, bisexual, trans, and queer). The PRMR analysis by urban–rural classifications shows that from 2016 to 2018, the PRMRs were lower in larger metro counties compared to the rural noncore counties (13.8 deaths per 100,000 live births for persons living in large fringe metro counties which were lower than 24.4 deaths per 100,000 live births for persons living in noncore counties) (22). Women who are uninsured or have Medicaid coverage living in rural areas, and living in counties with lower income and educational attainment, had to travel farther to the nearest hospital with obstetric services or neonatal care than their counterparts (23). This disparity of access to care leads to insufficient care in high-risk pregnancies (24) likely leading to worsened perinatal outcomes. Compared to heterosexual women, bisexual and lesbian women were more likely to report miscarriages (OR = 1.77, 95% CI = 1.34, 2.35) and pregnancies ending in stillbirth (OR = 2.85, 95% CI = 1.40, 5.83). Lesbian women were also more likely to report low birth weight infants (OR = 2.64, 95% CI = 1.38, 5.07), and bisexual and lesbian women were more likely to report very preterm births (OR = 1.84, 95% CI = 1.11, 3.04) compared to heterosexual women (25). Patients with a SUD history also face implicit bias in their treatment. A study explored potential stigma and attitudes among medical providers within a maternal/fetal healthcare setting toward maternal SUD (26), and it was found that providers who consider drug abuse as a disease rather than a stigma have more favorable interactions with obstetric patients with a drug abuse history.

### Disparities in obstetric neurocritical care

#### Cerebrovascular disease: Ischemic stroke, cerebral venous sinus thrombosis, and hemorrhagic stroke

Stroke is a leading cause of morbidity and mortality around the world, as well as in the US. It is usually grouped into categories of ischemic stroke (IS) and hemorrhagic stroke (HS), with cerebral venous sinus thrombosis (CVST) variably discussed separately in the literature. In the US, data from 2015 to 2018 report a total of 7.6 million patients over age 20 with stroke over the course of those 3 years (27). Of those, 4.1 million were female patients, compared to 3.5 million male patients. The lifetime risk of stroke for women is one in five, and one in six for men. A study in the Netherlands showed an increase in the incidence of stroke from 1998 to 2010 (28). According to their data in 2010 for women aged 18–49, within the childbearing age, the incidence of stroke was 18.86 per 100,000 person-years, compared to 15.64 in their male counterparts. When broken down for type of stroke (IS vs. hemorrhagic vs. any type which included CVST), the difference in incidence between women and men was statistically significant in any type and IS categories but not significant in HS. They referenced the possibility that female-specific factors could play a role in this difference, pregnancy being one of those factors.

For women of childbearing age, the overall incidence of stroke of all types is reported as 11 per 100,000 (29). In a meta-analysis reviewing the incidence of stroke in pregnancy, the pooled data from studies across the world showed an all-type stroke incidence of 30 per 100,000 live births (30). These data ranged from regions with higher incidence such as North America, Taiwan, and India, to lower incidence countries such as Israel. In the US, an incidence of 38.6 per 100,000 live births has been reported (31). The data suggest an increase of three times the stroke incidence in pregnancy compared to all strokes during childbearing age with concern that this is still an underestimated number.

Maternal stroke and maternal stroke-associated in-hospital mortality have been found to be higher among Black women (32). In a study analyzing the National Inpatient Sample (NIS) 2000–2001 (33), pregnant Black women were found to have the highest risk of stroke (52.5 per 100,000 deliveries) compared to Hispanic women or White women. It has been shown that maternal morbidity and mortality are higher among Black and Hispanic mothers compared to White mothers when it comes to hypertensive disorders of pregnancy (34–36), while among those with chronic hypertension, all women who were in the minority population including Asian and Pacific Islanders had higher stroke risk compared to White women (36). Globally, the geographic disparity in stroke event rate was reported (21), and it showed variability among studies from North America, Taiwan, India, and Israel. This geographic disparity can be attributed to variability in stroke risk factors, access, and quality of obstetric care, the consequence of which can translate to a higher prevalence of cerebrovascular diseases.

Hemorrhagic stroke is the most common stroke subtype in patients who are pregnant, compared to the general population where IS is more common (37). It is also responsible for 5%−12% of all maternal deaths with high mortality rates of 35%−83% (38). The hypertensive disorders of pregnancy are reported to be responsible for the highest risk of maternal HS. The most common reason for preeclampsia-associated deaths was ICH (36–40). These disorders occur with co-existent racial disparities and a continued lack of improvement, as the rate of these disorders continues to climb (40–43). In terms of HS, a study conducted in Japan found a high HS rate (73.5%) among patients with pregnancy-associated strokes (41) which is consistent with reports that Asians have an increased risk of the hemorrhagic type of stroke compared with Caucasians and African Americans (42). However, in a study analyzing the NIS for the years 1993 through 2002, it was found that African American race was an independent risk factor for ICH in pregnancy (39). In addition to this, a study utilizing administrative data from New York, California, and Florida showed consistent findings that non-White race including African Americans, Asians, and Hispanics, was an independent predictor of ICH among patients who are pregnant (43). When analyzing the trends in spontaneous SAH in pregnancy in a 12-year period, it was found that the greatest increase in the incidence of SAH was among African American women (13.4–16.39 per 100,000 births) (44). In this population, it was also shown that pregnant women who were admitted to hospitals with larger bed sizes or urban teaching hospitals had lower odds of discharge to long-term facilities.

The above represents alarming data, and with stroke incidence on the rise overall, there should be a call to action to address this special population of patients, with attention to higher incidence countries and regions and modifiable factors, as well as research to determine causes for this increased risk and possible treatment mechanisms. It is also important to specify ways to acknowledge and improve the racial/ethnic and other disparities that exist within this subset of the population.

#### Post-cardiac arrest

Cardiac arrest is a rare but feared complication in pregnancy. The rates of out-of-hospital cardiac arrest in the general US population is 95/100,000, 67–170/100,000 in Europe, and 60/100,000 in South Korea, with poor outcomes. In-hospital cardiac arrest rates are 6–7/1,000 in US and 1.5–2.8/1,000 admissions in Europe (45). Taking a closer look at younger patients, the rate of sudden cardiac arrest in one study was reported as 4.4/100,000 in patients 25–35 and 1.44/100,000 in patients 14–24, of all genders combined (46). The prevalence of maternal cardiac arrest suggested in one review (47) was one in 12,000 in the US and one in 16,000 in the UK, comparable to 8.3 and 6.25 per 100,000, respectively, significantly higher than rates of the general population around childbearing age. Only minimal data are available to give information on specific disparities of care among patients with maternal post-cardiac arrest neurologic morbidity. In a study in California analyzing 64 cardiovascular pregnancy-related deaths, they found that women were likely to be African American if they died from cardiovascular disease (48). The implications of this for post-cardiac arrest care cannot be understated. Guidelines that involve neurologic resuscitation post-cardiac arrest should include patients who are pregnant (49–51).

#### Traumatic brain injury

Traumatic brain injury is a global health crisis, with over 27 M people worldwide experiencing TBI in 2016 alone, along with over 55 M people living with disability related to prior TBI. There are numerous literature reviews on TBI in the general population but few focused on TBI in pregnancy. The rate of pregnancy-associated trauma has been reported to be 6%−46% (52), creating a high risk for morbidity and mortality. A study on women who had moderate–severe TBI showed that fewer patients with TBI had one or more live births and had more post-partum issues compared to non-TBI controls (53). It was noted that in TBI across a woman's lifespan, women have the highest incidence of TBI in ages 21–50, encompassing the childbearing age (54). The difference in mechanisms was noted where TBI in women was from falls, collisions, and domestic violence unlike in men, where it is from motor vehicle accidents or combat. TBI is also associated with pregnancy difficulties (55). Intimate partner violence (IPV) that can result in TBI significantly affects ethnic minorities (Black/African American, Hispanic/Latina, Native American/Alaska Native, and Asian American) (56), and TBI from IPV has been found to result in chronic psychiatric disorders such as posttraumatic stress disorder (57) and post-partum depression (58). This knowledge is important when treating a patient who is pregnant in the minority population with severe TBI in neurocritical care.

#### Status epilepticus

Status epilepticus has limited literature in regard to disparities in care among the obstetric population. It is generally known that seizures are well-controlled even during pregnancy (59). However, 30%−40% of patients with epilepsy can still have drug-resistant epilepsy and pregnancy may complicate their management (60). A study analyzing only pregnant patients with refractory epilepsy found that 8.5% of the patients developed SE over the course of the pregnancy which is higher than 0.6%−2.1%, which has been reported prior (61, 62). Low compliance is one of the factors that was associated with SE (59). Poor compliance has been shown to be associated with lower socioeconomic status and inadequate insurance (63). Few studies on pregnancy outcomes and seizures in terms of race/ethnicity have not been conclusive due to the small sample and lower prevalence in this specific population (64, 65). Studies on SE associated with pregnancy and disparities in care are significantly lacking. There is a need for continued focus on this patient population when it comes to SE as well.

### Future steps and directions to eliminate disparities in obstetric neurocritical care

Disparities in maternal healthcare have been well reported; however, there is little sign that the disparities are dissipating. The summary of the disparities found in obstetric and neurocritical care is shown (Table 1). These factors may be considered in the care of neurocritically-ill pregnant patients since these explore the overlapping risk factors for neurocritically-ill pregnant patients that can affect acute neurologic disease states and their outcomes.

**Table 1:** Summary of areas of disparities in obstetric care and neurocritical care.

**Obstetric maternal care**		**Neurocritical care**	
**Condition/outcome of interest**	**Disparities of care**	**Condition/outcome of interest**	**Disparities of care**
Maternal mortality rate	- Black women 3–4 × than white women - Higher mortality rates among those living in rural vs. urban areas	Maternal stroke	- Higher in Black women - Those with chronic hypertension, all those from the minority population had greater stroke risk compared to white women
Hypertension in pregnancy	- More common in black compared to white	Maternal stroke-associated mortality	- Higher in Black women
Cardiomyopathy	- More common in black compared to white	Hypertensive disorders of pregnancy	- Black and Hispanic have higher morbidity and mortality compared to white mothers
Thromboembolic complications	- More common in black compared to white	Hemorrhagic stroke	- Asians have increased risk of ICH vs. white and African American mothers - Non-white race an independent predictor of ICH among pregnant patients
General obstetric comorbidity	- Higher occurrence among the uninsured and those on Medicaid	SAH in pregnancy	- Greatest increase in incidence in 12-years were among African-Americans - Larger bed size or urban teaching hospitals had lower odds of discharge to long-term facilities
Miscarriage rate and stillbirth	- Lesbian and bisexual women were more likely to report miscarriage, still birth and preterm births	Cardiac arrest in pregnancy	- Cardiovascular pregnancy-related deaths are likely to be African-Americans than White
Post-partum hemorrhage	- Higher rate among mother-mother partnerships	Traumatic brain injury in pregnancy	Intimate partner violence significantly affects ethnic minorities
		Status epilepticus in pregnancy	Low compliance (associated with lower socioeconomic status and inadequate insurance) affects development of status epilepticus during pregnancy

ICH, intracerebral hemorrhage; SAH, subarachnoid hemorrhage.

**Figure 1:**
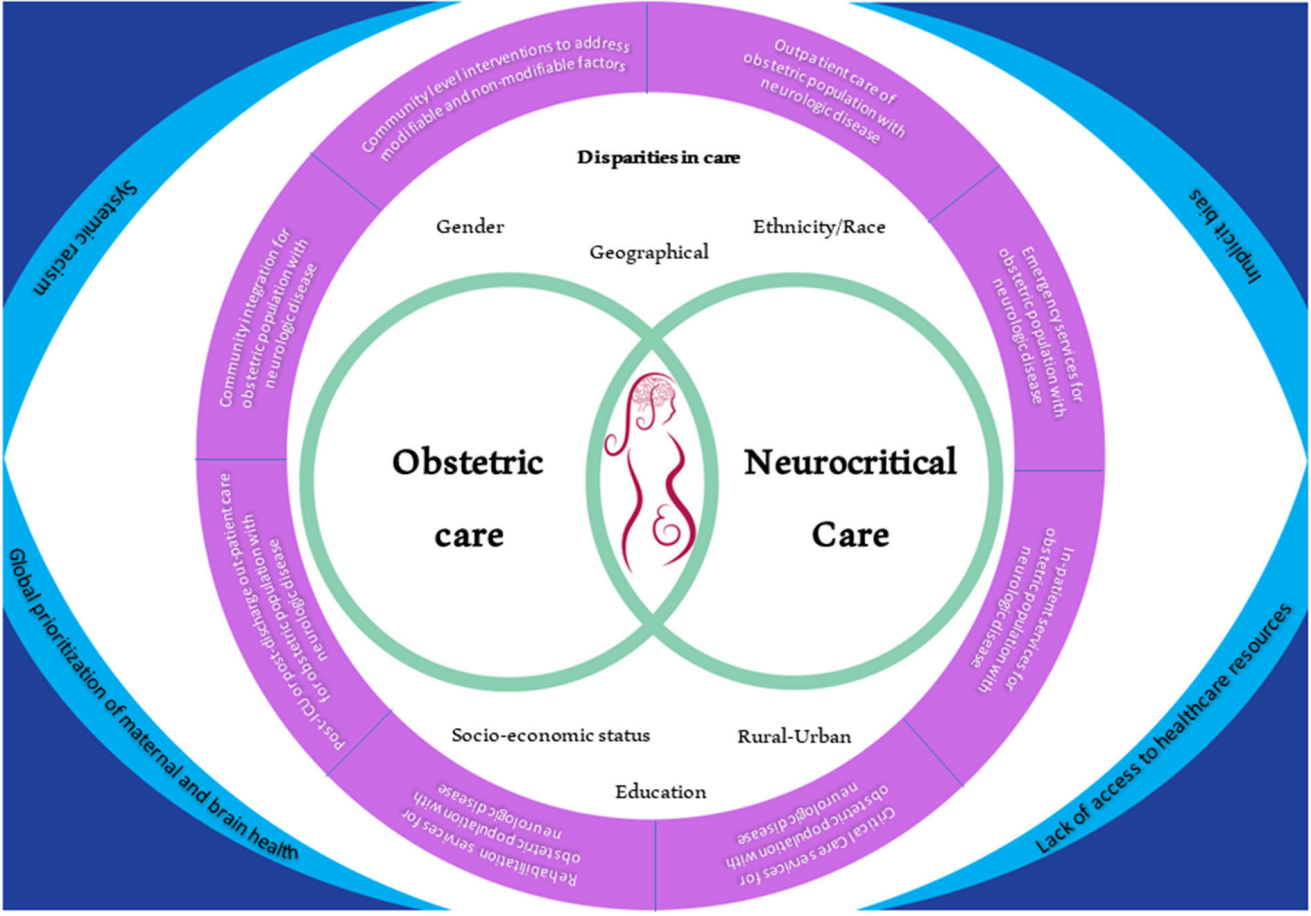
A new lens into looking at the landscape of disparities in obstetric neurocritical care and its integration into the patient continuum of care. On the corners are the main issues that are prevalent and that have to be integrated across the continuum of care. All of these issues also affect both obstetric care and neurocritical care and ultimately affect the outcome of the mother and the fetus. The areas of disparities in care that should be considered through the care of these patients are also shown.

Figure 1 shows how clinicians, policymakers, organizational leaders, and patients can potentially approach the challenge caused by the disparities in obstetric and neurocritical care along the continuum of care. Table 2 shows potential actionable solutions that can promote the decrease in disparities of care at the institutional, regional, national, and global levels. Leveraging organizational resources that can provide sets of bundled guidance to provide standardization for maternal healthcare services can be helpful (16). Such standardization can likely help address structural racism (66) that is prevalent in the healthcare industry due to pre-existing economic and social barriers (67). Collaboration among national and international societies should also be done to broaden the outreach for research, education, and information sharing among clinicians. One example of this is the United Kingdom registry of high-risk obstetric anesthesia with a focus on neurologic disease which collected data from 1997 to 2002 (68). Such a model entailed organization members to report, *via* a data collection form, any neurologic cases that they encountered in their obstetric units.

**Table 2:** Potential targeted and actionable interventions to address disparities in obstetric care and neurocritical care of pregnant patients who are neurotically ill.

**Institutional**	**Regional**	**National**	**Global**
Educational curriculum on implicit bias, systemic racism and cultural competency	Policy prioritization at the state, country and global public health levels	Establish and coordinate national and global multicenter registries on obstetric and neurocritical care outcomes	
Simulation and virtual reality incorporation for competency testing	funding for improving access in obstetric care and neurocritical care	Inter-organization and inter-societal collaboration on advocacy and initiatives (ACOG, SMFM, NCS, AAN, ANA, AES, BTF)	Global public health prioritization through the World Health Organization advocacy
Institutional nurse navigator for the neurocritically-ill patient with prioritization of those considered a minority	Interprofessional collaboration for education and research	Federal government advocacy for prioritization of obstetric neurocritical care issues	Collaboration of international societies to establish guidance of care accounting for disparities globally
	Telehealth use with collaboration between neurocritical care and maternal fetal medicine	Advance policies to safely be able to integrate pregnant patients in research	
	Inter-hospital agreements and affiliations for neurocritically-ill pregnant patients		

ACOG, American College of Obstetricians and Gynecologists; SMFM, Society for Maternal-Fetal Medicine; NCS, Neurocritical Care Society; AAN, American Academy of Neurology; ANA, American Neurological Association; AES, American Epilepsy Society; BTF, Brain Trauma Foundation.

Research has shown that nearly half of these maternal deaths and morbidity events are preventable by good quality healthcare services from preconception up until delivery. Attempts should be made to improve healthcare delivery to the underserved population possibly utilizing telehealth in collaboration with NCC and obstetric services (69–71). Obstetric tele-neurology has been explored globally as a means to bridge the gap to access; however, more robust data on metrics as well as optimization of available technological devices are needed (70). There are those with no access to the Internet that may benefit from telehealth services by collaborating with their local medical team closer to their home, an example of which is a telemedicine program in rural Panama (71). This type of undertaking requires not only healthcare support but also government support as well to be able to initiate and sustain this program.

The conceptual model approach to disparities in obstetric care has been explored before (1). The patient's socioeconomic status, age, ethnicity, race, education, marital status, insurance, language, employment, psychosocial support, and literacy all play a role in patient disparity analysis in addition to community, provider, and system factors.

There is a paucity of data on the best way to approach disparities of care in obstetric neurocritical care. Novel to this review, the major gaps in research in disparities of care among the obstetric neurocritically-ill population were delineated. The possible directions to take to move toward decreasing disparities in care for this population were also described.

## Conclusion

Disparities in the care of the obstetric population affect maternal outcomes. Due to the increased risk of acute neurologic comorbidities and the risk of concomitant acute neurologic conditions among the obstetric population, the effect of these disparities on the management and outcomes of the patient who is neurocritically-ill should be studied. There is a large gap in information regarding disparities in the care of obstetric neurocritically-ill patients. Further research should be done to delineate this as part of the multidimensional approach to eliminate the disparities in obstetric and neurocritical care.

## Author contributions

SM, DA, RO, and FI: manuscript writing. CN: conceptualization, manuscript writing, and final editing. All authors contributed to the article and approved the submitted version.

## References

[1] Howell EA. Reducing disparities in severe maternal morbidity and mortality. Clin Obstet Gynecol. (2018) 61:387–99. 10.1097/GRF.0000000000000349 29346121 PMC5915910

[2] Small MJ Allen TK Brown HL. Global disparities in maternal morbidity and mortality. Semin Perinatol. (2017) 41:318–22. 10.1053/j.semperi.2017.04.009 28669415 PMC5608036

[3] Howell EA Zeitlin J. Improving hospital quality to reduce disparities in severe maternal morbidity and mortality. Semin Perinatol. (2017) 41:266–72. 10.1053/j.semperi.2017.04.002 28735811 PMC5592149

[4] Ijäs P. Trends in the incidence and risk factors of pregnancy-associated stroke. Front Neurol. (2022) 13:833215. 10.3389/fneur.2022.833215 35481266 PMC9035801

[5] Miller EC Vollbracht S. Neurology of preeclampsia and related disorders: an update in neuro-obstetrics. Curr Pain Headache Rep. (2021) 25:40. 10.1007/s11916-021-00958-z 33825997 PMC10069269

[6] Keepanasseril A Pfaller B Metcalfe A Siu SC Davis MB Silversides CK. Cardiovascular deaths in pregnancy: growing concerns and preventive strategies. Can J Cardiol. (2021) 37:1969–78. 10.1016/j.cjca.2021.09.022 34600086

[7] Howell EA Zeitlin J. Quality of care and disparities in obstetrics. Obstet Gynecol Clin North Am. (2017) 44:13–25. 10.1016/j.ogc.2016.10.002 28160890 PMC5300700

[8] Creanga AA Bateman BT Kuklina EV Callaghan WM. Racial and ethnic disparities in severe maternal morbidity: a multistate analysis, 2008-2010. Am J Obstet Gynecol. (2014) 210:435. 10.1016/j.ajog.2013.11.039 24295922

[9] Lin C Arevalo YA Nanavati HD Lin DM. Racial differences and an increased systemic inflammatory response are seen in patients with COVID-19 and ischemic stroke. Brain Behav Immun Health. (2020) 8:100137. 10.1016/j.bbih.2020.100137 32904928 PMC7462566

[10] Sisti G Faraci A Silva J Upadhyay R. Neutrophil-to-lymphocyte ratio, platelet-to-lymphocyte ratio, and routine complete blood count components in HELLP syndrome: a matched case control study. Medicina. (2019) 55:123. 10.3390/medicina55050123 31072037 PMC6572204

[11] Khanzadeh S Tahernia H Hernandez J Sarcone C Lucke-Wold B Salimi A. Predictive role of neutrophil to lymphocyte ratio in adnexal torsion: a systematic review and meta-analysis. Mediators Inflamm. (2022) 2022:1–8. 10.1155/2022/9680591 36387932 PMC9649322

[12] Hai L Hu ZD. The clinical utility of neutrophil to lymphocyte ratio in pregnancy related complications: a mini-review. J Lab Precis Med. (2020) 5:1–9. 10.21037/jlpm.2019.10.03

[13] Azab B Camacho-Rivera M Taioli E. Average values and racial differences of neutrophil lymphocyte ratio among a nationally representative sample of United States subjects. PLoS ONE. (2014) 9:e112361. 10.1371/journal.pone.0112361 25375150 PMC4223021

[14] Taşkömür AT Erten Ö. The role of cystatin C, neutrophil-lymphocyte ratio and platelet-lymphocyte ratio in the evaluation of kidney function in women with preeclampsia. Taiwan J Obstet Gynecol. (2021) 60:615–20. 10.1016/j.tjog.2021.05.007 34247797

[15] Willman M Willman J Lucke-Wold B. Endocrine resistant breast cancer: brain metastasis. Explor Target Antitumor Ther. (2022) 3:240–51. 10.37349/etat.2022.00081 35505937 PMC9060566

[16] Frisk G Svensson T Bäcklund LM Lidbrink E Blomqvist P Smedby KE. Incidence and time trends of brain metastases admissions among breast cancer patients in Sweden. Br J Cancer. (2012) 106:1850–3. 10.1038/bjc.2012.163 22531629 PMC3364124

[17] Rauscher GH Silva A Pauls H Frasor J Bonini MG Hoskins K. Racial disparity in survival from estrogen and progesterone receptor-positive breast cancer: implications for reducing breast cancer mortality disparities. Breast Cancer Res Treat. (2017) 163:321–30. 10.1007/s10549-017-4166-z 28251385 PMC5410404

[18] Proskynitopoulos PJ Lam FC Sharma S Young BC Laviv Y Kasper EM. A review of the neurosurgical management of brain metastases during pregnancy. Can J Neurol Sci. (2021) 48:698–707. 10.1017/cjn.2020.254 33213549 PMC8527832

[19] Sharma A Nguyen HS Lozen A Sharma A Mueller W. Brain metastases from breast cancer during pregnancy. Surg Neurol Int. (2016) 7:S603–6. 10.4103/2152-7806.189730 27656319 PMC5025954

[20] Howell EA Ahmed ZN. Eight steps for narrowing the maternal health disparity gap. Contemp Ob Gyn. (2019) 64:30–6. 31673195 PMC6822100

[21] Hall JA Benton L Copas A Stephenson J. Pregnancy intention and pregnancy outcome: systematic review and meta-analysis. Matern Child Health J. (2017) 21:1051–63. 10.1007/s10995-016-2237-0 28093686 PMC5357274

[22] Centers for Disease Control and Prevention. Pregnancy Mortality Surveillance System. Maternal and Infant Health | CDC. Atlanta, GA: Reproductive Health. (2019).

[23] Hung P Casey MM Kozhimannil KB Karaca-Mandic P Moscovice IS. Rural-urban differences in access to hospital obstetric and neonatal care: how far is the closest one? J Perinatol. (2018) 38:645–52. 10.1038/s41372-018-0063-5 29453436

[24] Phiri SNA Fylkesnes K Moland KM Byskov J Kiserud T. Rural-urban inequity in unmet obstetric needs and functionality of emergency obstetric care services in a Zambian district. PLoS ONE. (2016) 11:e0145196. 10.1371/journal.pone.0145196 26824599 PMC4732684

[25] Everett BG Kominiarek MA Mollborn S Adkins DE Hughes TL. Sexual orientation disparities in pregnancy and infant outcomes. Matern Child Health J. (2019) 23:72–81. 10.1007/s10995-018-2595-x 30019158 PMC6501574

[26] Weber A Miskle B Lynch A Arndt S Acion L. Substance use in pregnancy: identifying stigma and improving care. Subst Abuse Rehabil. (2021) 12:105–21. 10.2147/SAR.S319180 34849047 PMC8627324

[27] CDC. Cdc.Gov Stroke Facts. Atlanta, GA: CDC (2020).

[28] Ekker MS Verhoeven JI Vaartjes I van Nieuwenhuizen KM Klijn CJM de Leeuw FE. Stroke incidence in young adults according to age, subtype, sex, and time trends. Neurology. (2019) 92:e2444–54. 10.1212/WNL.0000000000007533 31019103

[29] Bushnell CD. Stroke in women: risk and prevention throughout the lifespan. Neurol Clin. (2008) 26:1161–76. 10.1016/j.ncl.2008.05.009 19026906 PMC2634299

[30] Swartz RH Cayley ML Foley N Ladhani NNN Leffert L Bushnell C. The incidence of pregnancy-related stroke: a systematic review and meta-analysis. Int J Stroke. (2017) 12:687–97. 10.1177/1747493017723271 28884652

[31] Leffert LR Clancy CR Bateman BT Bryant AS Kuklina EV. Hypertensive disorders and pregnancy-related stroke : frequency, trends, risk factors, and outcomes. Obstet Gynecol. (2015) 125:124–31. 10.1097/AOG.0000000000000590 25560114 PMC4445352

[32] Elgendy IY Gad MM Mahmoud AN Keeley EC Pepine CJ. Acute stroke during pregnancy and puerperium. J Am Coll Cardiol. (2020) 75:180–90. 10.1016/j.jacc.2019.10.056 31948647

[33] James AH Bushnell CD Jamison MG Myers ER. Incidence and risk factors for stroke in pregnancy and the puerperium. Obstet Gynecol. (2005) 106:509–16. 10.1097/01.AOG.0000172428.78411.b0 16135580

[34] Creanga AA Berg CJ Syverson C Seed K Bruce FC Callaghan WM. Race, ethnicity, and nativity differentials in pregnancy-related mortality in the united states: 1993-2006. Obstet Gynecol. (2012) 120:261–8. 10.1097/AOG.0b013e31825cb87a 22825083

[35] Persky RW Turtzo LC McCullough LD. Stroke in women: disparities and outcomes. Curr Cardiol Rep. (2010) 12:6–13. 10.1007/s11886-009-0080-2 20425178 PMC2861793

[36] Miller EC Zambrano Espinoza MD Huang Y Friedman AM Boehme AK Bello NA. Maternal race/ethnicity, hypertension, and risk for stroke during delivery Admission. J Am Heart Assoc. (2020) 9:e014775. 10.1161/JAHA.119.014775 31973601 PMC7033883

[37] Liang ZW Lin L Gao WL Feng LM. A clinical characteristic analysis of pregnancy-associated intracranial haemorrhage in China. Sci Rep. (2015) 5:9509. 10.1038/srep09509 25819941 PMC4377582

[38] Foo L Bewley S Rudd A. Maternal death from stroke: a thirty year national retrospective review. Eur J Obstet Gynecol Reprod Biol. (2013) 171:266–70. 10.1016/j.ejogrb.2013.09.021 24128926

[39] Bateman BT Schumacher HC Bushnell CD Pile-Spellman J Simpson LL Sacco RL. Intracerebral hemorrhage in pregnancy: frequency, risk factors, and outcome. Neurology. (2006) 67:424–9. 10.1212/01.wnl.0000228277.84760.a2 16894102

[40] Cordonnier C Sprigg N Sandset EC Pavlovic A Sunnerhagen KS Caso V. Stroke in women-from evidence to inequalities. Nat Rev Neurol. (2017) 13:521–32. 10.1038/nrneurol.2017.95 28731036

[41] Yoshida K Takahashi JC Takenobu Y Suzuki N Ogawa A Miyamoto S. Strokes associated with pregnancy and puerperium: a Nationwide Study by the Japan stroke society. Stroke. (2017) 48:276–82. 10.1161/STROKEAHA.116.014406 28028148

[42] Sells CM Feske SK. Stroke in pregnancy. Semin Neurol. (2017) 37:669–78. 10.1055/s-0037-1608940 29270940

[43] Meeks JR Bambhroliya AB Alex KM Sheth SA Savitz SI Miller EC. Association of primary intracerebral hemorrhage with pregnancy and the postpartum period. JAMA Netw Open. (2020) 3:e202769. 10.1001/jamanetworkopen.2020.2769 32286658 PMC7156993

[44] Limaye K Patel A Dave M Kenmuir C Lahoti S Jadhav AP. Secular increases in spontaneous subarachnoid hemorrhage during pregnancy: a nationwide sample analysis. J Stroke Cerebrovasc Dis. (2019) 28:1141–8. 10.1016/j.jstrokecerebrovasdis.2019.01.025 30711414

[45] Lee S Lee SW Han KS Ki M Ko YH Kim SJ. Analysis of characteristics and mortality in cardiac arrest patients by hospital level: a nationwide population-based study. J Korean Med Sci. (2021) 36:e173. 10.3346/jkms.2021.36.e173 34184437 PMC8239426

[46] Meyer L Stubbs B Fahrenbruch C Maeda C Harmon K Eisenberg M. Incidence, causes, and survival trends from cardiovascular-related sudden cardiac arrest in children and young adults 0 to 35 years of age: a 30-year review. Circulation. (2012) 126:1363–72. 10.1161/CIRCULATIONAHA.111.076810 22887927

[47] Zelop CM Einav S Mhyre JM Martin S. Cardiac arrest during pregnancy: ongoing clinical conundrum. Am J Obstet Gynecol. (2018) 219:52–61. 10.1016/j.ajog.2017.12.232 29305251

[48] Hameed AB Lawton ES McCain CL Morton CH Mitchell C Main EK. Pregnancy-related cardiovascular deaths in California: beyond peripartum cardiomyopathy. Am J Obstet Gynecol. (2015) 213:379. 10.1016/j.ajog.2015.05.008 25979616

[49] Geocadin RG Wijdicks E Armstrong MJ Damian M Mayer SA Ornato JP. Practice guideline summary: reducing brain injury following cardiopulmonary resuscitation. Neurology. (2017) 88:2141–9. 10.1212/WNL.0000000000003966 28490655 PMC5447399

[50] Berg KM Cheng A Panchal AR Topjian AA Aziz K Bhanji F. Part 7: systems of care 2020 American heart association guidelines for cardiopulmonary resuscitation and emergency cardiovascular care. Circulation. (2020) 142:S580–604. 10.1161/CIR.0000000000000918 33081524

[51] Nolan JP Sandroni C Böttiger BW Cariou A Cronberg T Friberg H. European Resuscitation Council and European Society of Intensive Care Medicine guidelines 2021: post-resuscitation care. Intensive Care Med. (2021) 47:369–421. 10.1007/s00134-021-06368-4 33765189 PMC7993077

[52] Chames MC Pearlman MD. Trauma during pregnancy: outcomes and clinical management. Clin Obstet Gynecol. (2008) 51:398–408. 10.1097/GRF.0b013e31816f2aa7 18463469

[53] Colantonio A Mar W Escobar M Yoshida K Velikonja D Rizoli S. Women's health outcomes after traumatic brain injury. J Womens Health. (2010) 19:1109–16. 10.1089/jwh.2009.1740 20469963

[54] Blaya MO Raval AP Bramlett HM. Traumatic brain injury in women across lifespan. Neurobiol Dis. (2022) 164:105613. 10.1016/j.nbd.2022.105613 34995753

[55] Vaajala M Kuitunen I Nyrhi L Ponkilainen V Kekki M Luoto T. Pregnancy and delivery after traumatic brain injury: a nationwide population-based cohort study in Finland. J Matern Fetal Neonatal Med. (2022) 25:9709–16. 10.1080/14767058.2022.2050899 35282782

[56] Stockman JK Hayashi H Campbell JC. Intimate partner violence and its health impact on ethnic minority women. J Womens Health. (2015) 24:62–79. 10.1089/jwh.2014.4879 25551432 PMC4302952

[57] Hunnicutt G Murray C Lundgren K Crowe A Olson L. Exploring correlates of probable traumatic brain injury among intimate partner violence survivors. J Aggress Maltreat Trauma. (2019) 28:677–94. 10.1080/10926771.2019.1587656

[58] Silveira MF Mesenburg MA Bertoldi AD de Mola CL Bassani DG Domingues MR. The association between disrespect and abuse of women during childbirth and postpartum depression: findings from the 2015 Pelotas birth cohort study. J Affect Disord. (2019) 256:441–7. 10.1016/j.jad.2019.06.016 31252237 PMC6880287

[59] Kusznir Vitturi B Barreto Cabral F Mella Cukiert C. Outcomes of pregnant women with refractory epilepsy. Seizure. (2019) 69:251–7. 10.1016/j.seizure.2019.05.009 31128468

[60] Battino D Tomson T Bonizzoni E Craig J Lindhout D Sabers A. Seizure control and treatment changes in pregnancy: observations from the EURAP epilepsy pregnancy registry. Epilepsia. (2013) 54:1621–7. 10.1111/epi.12302 23848605

[61] Lu YT Hsu CW Tsai WC Cheng MY Shih FY Fu TY. Status epilepticus associated with pregnancy: a cohort study. Epilepsy Behav. (2016) 59:92–7. 10.1016/j.yebeh.2016.03.034 27116537

[62] Rajiv KR Radhakrishnan A. Status epilepticus in pregnancy: etiology, management, and clinical outcomes. Epilepsy Behav. (2017) 76:114–9. 10.1016/j.yebeh.2017.07.002 28899640

[63] Burneo JG Jette N Theodore W Begley C Parko K Thurman DJ. Disparities in epilepsy: report of a systematic review by the North American Commission of the international league against epilepsy. Epilepsia. (2009) 50:2285–95. 10.1111/j.1528-1167.2009.02282.x 19732134 PMC3181115

[64] Lawn N Laich E Ho S Martin R Faught E Knowlton R. Eclampsia, hippocampal sclerosis, and temporal lobe epilepsy: accident or association? Neurology. (2004) 62:1352–6. 10.1212/01.WNL.0000120544.64972.10 15111673

[65] Yerby M Koepsell T Daling J. Pregnancy complications and outcomes in a cohort of women with epilepsy. Epilepsia. (1985) 26:631–5. 10.1111/j.1528-1157.1985.tb05703.x 3841052

[66] Linnander EL Ayedun A Boatright D Ackerman-Barger K Morgenthaler TI Ray N. Mitigating structural racism to reduce inequities in sepsis outcomes: a mixed methods, longitudinal intervention study. BMC Health Serv Res. (2022) 22:975. 10.1186/s12913-022-08331-5 35907839 PMC9338573

[67] Johnson-Agbakwu CE Ali NS Oxford CM Wingo S Manin E Coonrod DV. Racism, COVID-19, and Health Inequity in the USA: a call to action. J Racial Ethn Health Disparities. (2022) 9:52–8. 10.1007/s40615-020-00928-y 33197038 PMC7668281

[68] May AE Fombon FN Francis S. UK registry of high-risk obstetric anaesthesia: report on neurological disease. Int J Obstet Anesth. (2008) 17:31–6. 10.1016/j.ijoa.2007.03.016 17981456

[69] Haranath S Ganapathy K Kesavarapu S Kuragayala S. eNeuroIntensive care in India: the need of the hour. Neurol India. (2021) 69:245–51. 10.4103/0028-3886.314591 33904432

[70] Alves DS Times VC da Silva ÉMA Melo PSA Novaes MA. Advances in obstetric telemonitoring: a systematic review. Int J Med Inform. (2020) 134:104004. 10.1016/j.ijmedinf.2019.104004 31816495

[71] Vega S Marciscano I Holcomb M Erps KA Major J Lopez AM. Testing a top-down strategy for establishing a sustainable telemedicine program in a developing country: the Arizona telemedicine program-US army-republic of panama initiative. Telemed J E Health. (2013) 19:746–53. 10.1089/tmj.2013.0025 23931731

